# Use of Long-Acting Injectable (LAI) aripiprazole during pregnancy A case series about baby and children neurodevelopment

**DOI:** 10.1192/j.eurpsy.2026.11121

**Published:** 2026-07-31

**Authors:** E. Sole, S. Andrés, C. Naranjo, C. Lopez, S. Lera, A. Roca-Lecumberri

**Affiliations:** Perinatal mental Health Unit. Psychiatry and clinical psychology Department. Hospital Clínic Barcelona., Barcelona, Spain

## Abstract

**Introduction:**

The perinatal period is associated with an increased risk of relapse in schizophrenia which can lead to long-term implications for the wellbeing of mother and baby. Long-Acting Injectable (LAI) antipsychotics have proven useful for relapse prevention in schizophrenia, but their use during pregnancy is still conflicting among clinical guidelines

**Objectives:**

We aimed at reporting a case series on the use of LAI aripiprazole during pregnancy in three women with schizophrenia during their pregnancies, with the derived obstetric neonatal and neurodevelopment outcomes. Informed consent to publish this case series was obtained from patients

**Methods:**

Description and review of neurodevelopment outcomes in babies exposed to LAI aripiprazole during pregnancy

**Results:**

We present three cases of pregnant women with schizophrenia. They are in treatment with LAI aripiprazole 400mg/28 days with clinical stability during their pregnancy. One patient presented two pregnancies with LAI aripiprazole treatment. Two of them didn’t take folic acid during the first trimester. Obstetric follow-up did not reveal obstetric complications or foetus malformations during both pregnancies. Patients maintained psychopathologic stability throughout pregnancies and postpartum periods. Bayley scale was evaluated, no adverse effects were reported in two babies, nevertheless, the other two presented mild-moderate deficit in social communication. After 6 years these two kids have psychological follow-up for probable autistic spectrum disorder.

**Conclusions:**

To our knowledge, these are the first reported cases of LAI aripiprazole treatment for schizophrenia to analyse the neurodevelopmental outcomes of children exposed until 6 years old of the children. In total, patients had four infants. Neither malformations nor obstetric complications in pregnancies were detected while patients were on monthly LAI aripiprazole. Moreover, children born to women with schizophrenia may be at risk for a variety of such adverse early developmental exposures. Prenatal exposures might be social (e.g. unemployment) behavioural (suboptimal eating, with folic acid or vitamin D deficiency and/or medical (diabetes, hypertension). Poor maternal nutrition is linked not only to folic acid deficiency, but also to vitamin D deficiency.

In conclusion, there is no clear evidence on reproductive safety and more studies that analyse children in the short and medium term are needed. Nevertheless, LAI aripiprazole may be a therapeutic option during pregnancy and the postpartum period in women with schizophrenia when the expected benefits outweigh the potential risks. Indeed, we recommend consider specific dietary- supplements treatments before and during pregnancy as well.

**Disclosure of Interest:**

None Declared

